# CNN-Based Page Segmentation and Object Classification for Counting Population in Ottoman Archival Documentation

**DOI:** 10.3390/jimaging6050032

**Published:** 2020-05-14

**Authors:** Yekta Said Can, M. Erdem Kabadayı

**Affiliations:** College of Social Sciences and Humanities, Koc University, Rumelifeneri Yolu, 34450 Sarıyer, Istanbul, Turkey; mkabadayi@ku.edu.tr

**Keywords:** page segmentation, historical document analysis, convolutional neural networks, Arabic script layout analysis

## Abstract

Historical document analysis systems gain importance with the increasing efforts in the digitalization of archives. Page segmentation and layout analysis are crucial steps for such systems. Errors in these steps will affect the outcome of handwritten text recognition and Optical Character Recognition (OCR) methods, which increase the importance of the page segmentation and layout analysis. Degradation of documents, digitization errors, and varying layout styles are the issues that complicate the segmentation of historical documents. The properties of Arabic scripts such as connected letters, ligatures, diacritics, and different writing styles make it even more challenging to process Arabic script historical documents. In this study, we developed an automatic system for counting registered individuals and assigning them to populated places by using a CNN-based architecture. To evaluate the performance of our system, we created a labeled dataset of registers obtained from the first wave of population registers of the Ottoman Empire held between the 1840s and 1860s. We achieved promising results for classifying different types of objects and counting the individuals and assigning them to populated places.

## 1. Introduction

Historical documents are valuable cultural resources that provide the examination of the historical, social, and economic aspects of the past. Their digitization also provides immediate access for researchers and the public to these archives. However, for maintenance reasons, access to them might not be possible or could be limited. Furthermore, we can analyze and infer new information from these documents after the digitalization processes. For digitalizing the historical documents, page segmentation of different areas is a critical process for further document analysis [1]. Example applications of historical document processing could be historical weather analysis [2], personnel record analysis [3], and digitization of music score images (OMR) [4]. Page segmentation techniques analyze the document by dividing the image into different regions such as backgrounds, texts, graphics, and decorations [5]. Historical document segmentation is more challenging because of the degradation of document images, digitization errors, and variable layout types. Therefore, it is difficult to segment them by applying projection-based or rule-based methods [5].

Page segmentation errors have a direct impact on the output of the Optical Character Recognition (OCR), which converts handwritten or printed text into digitized characters. Therefore, page segmentation techniques for historical documents become important for the correct digitization. We can examine the literature on page segmentation under three subcategories [5]. The first category is the granular-based techniques, which combine the pixels and fundamental elements into large components [6,7,8]. The second category is the block-based techniques that divide the pages into small regions and then combine them into large homogenous areas [9,10]. The last one is the texture-based methods, which extract textual features classifying objects with different labels [11,12,13]. Except for the block-based techniques, these methods work in a bottom-up manner. The bottom-up mechanisms have better performance with documents in variable layout formats [14]. However, they are expensive in terms of computational power because there are plenty of pixels or small elements to classify and connect. Still, the advancement of the technology of CPUs and GPUs alleviates this burden. Feature extraction and classifier algorithm design are very crucial for the performance of page segmentation methods. Although document image analysis started with more traditional machine learning classifiers, with the emergence of Convolutional Neural Networks (CNNs), they are commonly used in the literature [4,5,15,16]. Convolutional neural networks can successfully capture the spatial relations in an image by applying relevant filters, which makes their performance better when compared to the traditional classifiers [17].

Arabic script is used in writing different languages, e.g., Ottoman, Arabic, Urdu, Kurdish, Persian. It could be written in different manners, which complicate the page segmentation procedure. It is a cursive script in which connected letters create ligatures [18]. Arabic words could further include dots and diacritics, which causes even more difficulties in the page segmentation.

In this study, we developed a software that automatically segments pages and recognizes objects for counting the population registered in Ottoman populated places. Our data came from the first population registers of the Ottoman Empire that were conducted in the 1840s. These registers were the result of an unprecedented administrative operation, which aimed to register each and every male subject of the empire, irrespective of age, ethnic or religious affiliation, or military or financial status. Therefore, they aimed to have universal coverage for the male populace, and thus, these registers can be called (proto-)censuses. The Ottoman state had registered selected segments of her population for tax and/or conscription purposes for centuries. The first universal population census covering the entire male and female population of the Ottoman Empire was conducted in the 1880s. Starting from the 1840s and for the very first time, all males irrespective of age, ethnicity, religion, or economic status were registered mainly for demographic reasons. This is the reason we call these registers proto-censuses. The geographical coverage of these registers is the entire Ottoman Empire in the mid-nineteenth Century, which encompassed the territories of around two dozen successor states of today in Southeast Europe and the Middle East. For this study, we are focusing on two locations: Nicaea in western Anatolia in Turkey and Svishtov, a Danubian town in Bulgaria.

In these censuses, officers prepared manuscripts without using hand-drawn or printed tables. Furthermore, there was not any pre-determined page structure. Page layouts could differ in different districts. There were also structural variations depending on the clerk. We created a labeled dataset to give as an input to the supervised learning algorithms. In this dataset, different regions and objects were marked with different colors. We then classified all pixels and connected the regions comprised of the same type of pixels. We recognized the populated place starting points and person objects on these unstructured handwritten pages and counted the number of people in all populated places and pages. Our system successfully counted the population in different populated places.

The structure of the remaining parts of the paper is as follows. In Section 2, the related work in historical document analysis will be reviewed. We describe the structure of the created database in Section 3. Our method for page segmentation and object recognition is described in Section 4. Experimental results and a discussion are presented in Section 5. We present the conclusion and future works of the study in Section 6.

## 2. Related Works

Document image analysis studies started in the early 1980s [19]. Laven et al. [20] developed a statistical learning-based page segmentation system. They created a dataset that included 932 page images of academic journals and labeled physical layout information manually. By using a logistic regression classifier, they achieved approximately 99% accuracy with 25 labels. The algorithm for segmentation was a variation of the XY-cut algorithm [21]. Arabic document layout analysis has also been studied with traditional algorithms in the literature. Hesham et al. [18] developed an automatic layout detection system for Arabic documents. They also added line segmentation support. After applying Sauvola binarization [22], noise filtering (Gaussian noise filter), and skewness correction algorithms (by using the Radon transform [23]), they classified text and non-text regions with the Support Vector Machine (SVM) algorithm. They further segmented lines and words.

In some cases, the historical documents might have a tabular structure, which makes it easier to analyze the layout. Zhang et al. [3] developed a system for analyzing Japanese Personnel Record 1956 (PR1956) documents, which included company information in a tabular structure. They segmented the document by using the text region with a complex tabular structure and applied Japanese OCR techniques to segmented images. Each document had five columns, and each column had a number of rows. Richarz et al. [2] also implemented a semi-supervised OCR system on historical weather reports with printed tables. They scanned 58 pages and applied segmentation by using the printed tables. Afterward, they recognized digits and seven letters in the document.

After the emergence of Neural Networks (NNs), NNs were also tested on Arabic document analysis systems. Bukhari et al. [8] developed an automatic layout detection system. The authors classified the main body and the side text by using the MultiLayer Perceptron (MLP) algorithm. They created a dataset consisting of 38 historical document images from a private library in the old city of Jerusalem. They achieved 95% classification accuracy. The convolutional neural network is also a type of deep neural network that can be used for most of the image processing applications [24]. CNN and Long Short-Term Memory (LSTM) were used for document layout analysis of scientific journal papers written in English in [25,26]. Amer et al. proposed a CNN-based document layout analysis system for Arabic newspapers and Arabic printed texts. They achieved approximately 90% accuracy in finding text and non-text regions.

CNNs are also used for segmenting historical documents. As mentioned previously, historical document analysis has challenges such as low image quality, degraded images, variable layouts, and digitization errors. The Arabic language also creates difficulties for document segmentation due to its cursive nature where letters are connected by forming ligatures. Words may also contain dots and diacritics, which could be problematic for segmentation algorithms. Although there are studies applying CNNs to historical documents [1,5,15], to the best of our knowledge, this study is the first to apply CNN-based segmentation and object recognition in historical handwritten Arabic script document analysis in the literature.

## 3. Structure of the Registers

Our case study focused on the registers of Nicaea and Svishtov district registers, with code names NFS.d. 1411, 1452, and NFS.d. 6314, respectively, available at the Turkish Presidency State Archives of the Republic of Turkey, Department of Ottoman Archives, in jpeg format, upon request. We aimed to develop a methodology to be implemented for an efficient distant reading of similar registers from various regions of the Empire prepared between the 1840s and the 1860s. As mentioned above, these registers provided detailed demographic information on male members of the households, i.e., names, family relations, ages, and occupations. Females in the households were not registered. The registers became available for research at the Ottoman State Archives in Turkey, as recently as 2011. Their total number is around 11,000. Until now, they have not been subject to any systematic study. Only individual registers were transliterated in a piecemeal manner. The digital images of the recordings were 2210 × 3000 pixels in size.

**Figure 1:**
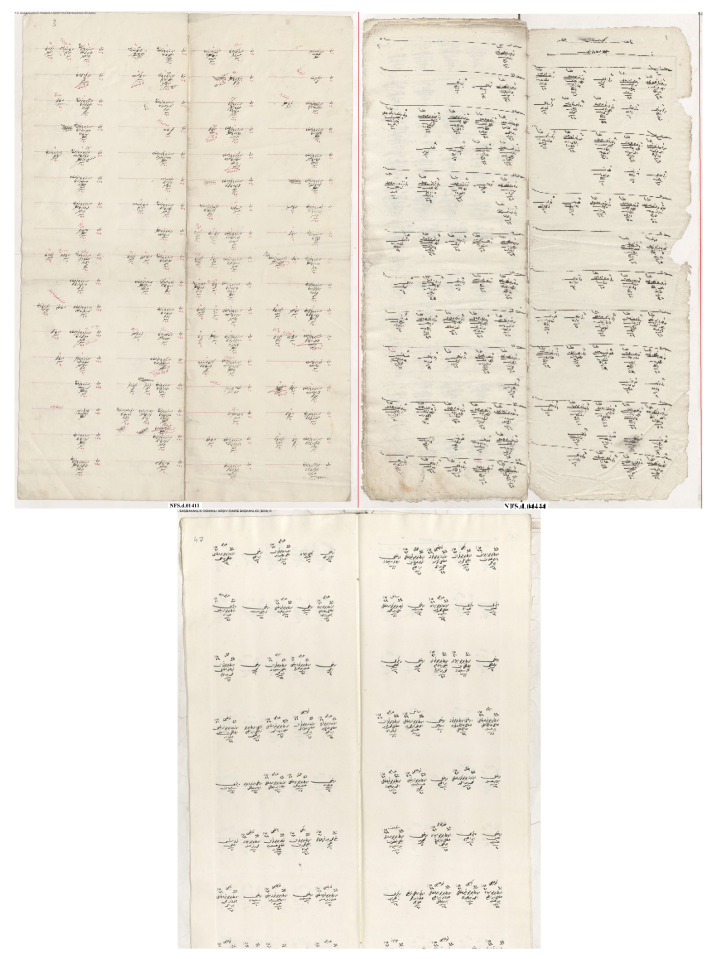
Three sample pages of the registers belonging to three different districts. The layout of pages can change between districts.

As mentioned previously, the layout of these registers could change from district to district (see Figure 1), which made our task more complicated. In some registers, there were lines between households; some districts used color in numerals and row and column numbers; and shapes could vary from district to district. For example in some registers, households were separated with lines. In another format, households were the same as individual objects with only one difference: in the first line, “household” was written in Arabic. Furthermore, there was no standard in coloring and the number of people per page. When the people density in a page was too high, objects were intertwined and hard to separate. Such differences made it difficult to develop one strategy that would work for information retrieval from all documents.

**Figure 2:**
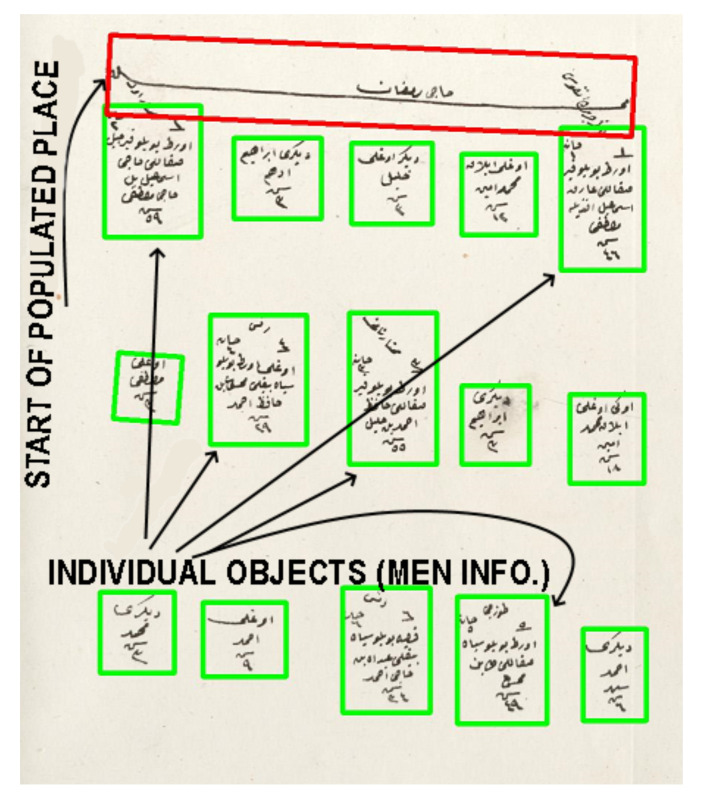
Start of the populated place (village or neighborhood) symbol and individual objects are demonstrated. When a new populated place is registered, its name is written at the top of a new page (populated place start symbol). Then, all men in this place are written one by one (individual objects). These objects include the name, age, appearance, and job of the individuals.

**Figure 3:**
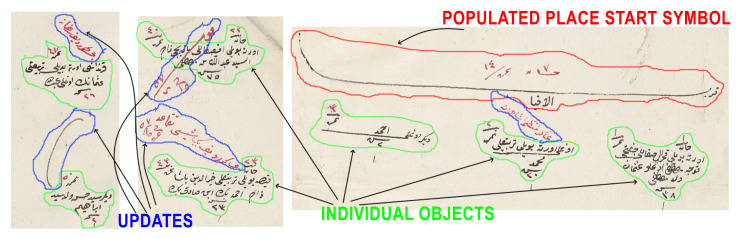
Example updates of registers are shown. Some of them can connect two individuals and can cause clustering errors. Green enclosed objects are individuals; red ones are populated place symbols; and blue ones are the updates connecting two other object types.

In this study, we worked with the generic properties of these documents. The first property was the populated place start symbol. This symbol was used in most of the districts and marked the start of the new populated place (see Figure 2). It included the name of the populated place (village or neighborhood). After this symbol, all men and their information were written one by one. They included demographic information (name, appearance, job, age, family relations) about the male citizens. There were also updates in these registers that marked the individuals when they went into the military service or died. The officers generally drew a line on the individual and sometimes mistakenly connected the individual with an adjacent one, which could cause some errors in the segmentation algorithm (see Figure 3).

## 4. Automatic Page Segmentation and Object Recognition System for Counting the Ottoman Population

### 4.1. Creating a Dataset

**Figure 4:**
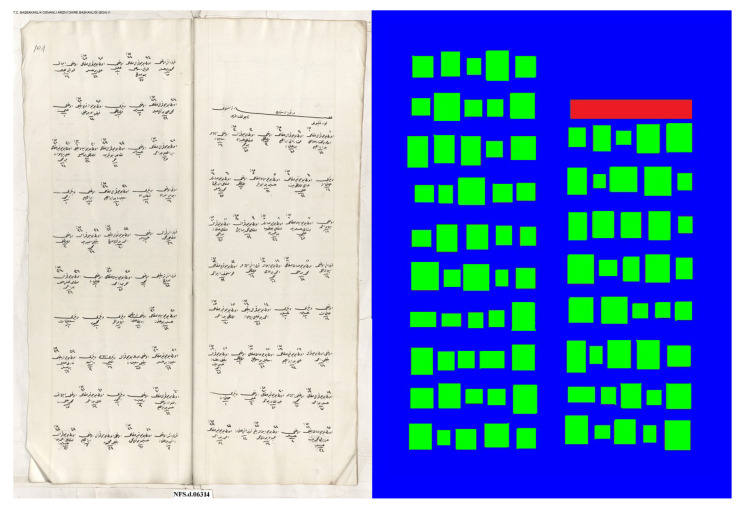
A sample register page and its labeled version are demonstrated. Different colors represent different object types. The background, which is the region between the objects and document borders, is marked with blue. The start of a populated place object is colored with red. The individual objects are marked with green.

To be able to use the dhSegment toolbox [15], we created a dataset with labels that belonged to three different classes. The first one was the background, which was the region between the objects and document borders. We marked this region as blue. The second one was the start of a populated place object, and we colored it with red. The last one was the individual registers, and we marked them with green. We marked 173 pages with the described labels. Fifty-one of them belonged to the Svishtov district, and one-hundred twenty-two of them belonged to the Nicaea district. An example of an original image and of labeled version are shown in Figure 4.

### 4.2. Training the CNN Architecture

In order to train a CNN for our system, we used the dhSegment toolbox [15]. This toolbox trains a system using the deep residual pretrained ResNet-50 architecture [27]. The toolbox has both a contracting path (follows the deep residual network in ResNet-50 [27]) and an expanding path, which maps low-resolution features to the original high-resolution features (see the terminology for expanding and contracting paths in [28]) [15]. The expanding path consisted of five blocks and a convolutional layer for pixel classification, and each deconvolution step consisted of upscaling of an image, concatenation of a feature map to a contracting one, 3 × 3 convolutional, and one ReLU layer.

**Figure 5:**
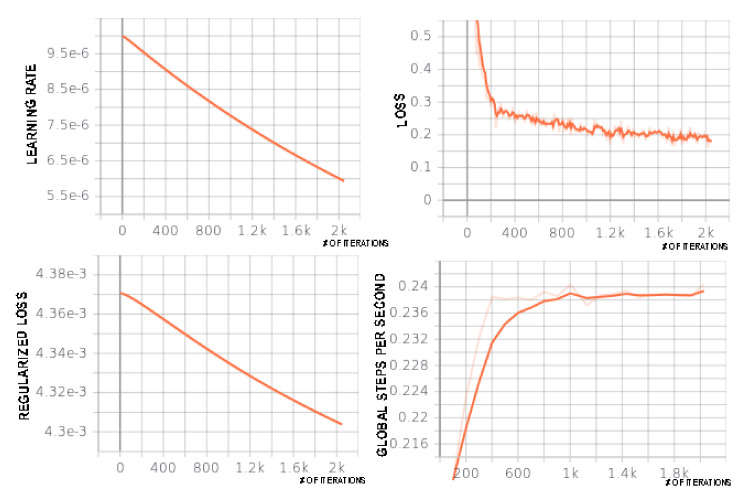
Training metrics are demonstrated. In the top left, the learning rate, in the top right, the loss function, in the bottom left, regularized loss, and in the bottom right, global steps per second metrics are demonstrated. The subfigures are created with Tensorboard. The horizontal axis is the increasing iterations.

In order to train the model, the toolbox used L2 regularization with $10^{-6}$ weight decay [15]. Xavier initialization [29] and the Adam optimizer [30] were applied. Batch renormalization [31] was employed to avoid a lack of diversity problem. The toolbox further downsized pictures and divided them into 300 to 300 patches for better fitting into the memory and providing support for training with batches. With the addition of margins, border effects were prevented. Because of the usage of pre-measured weights in the network, the training time was decreased substantially [15]. The training process exploited a variety of on-the-fly data augmentation techniques like rotation (from −0.2 to 0.2 rad), scaling (coefficient from 0.8 to 1.2), and mirroring. The system output the probabilities of each pixel belonging to one of the trained object types. Detailed metrics of one of the trained models by the integration of Tensorboard are shown in Figure 5.

We used a CPU for training a model. Training a model with 100 images took approximately 7 h. Testing an image lasted for 8.35 s on average.

### 4.3. Preparing the Dataset for Evaluation

We trained five different models for evaluating the performance of our system. Two models were trained with a register of one district and tested with a completely different district’s register. The other two models were trained and tested with the registers from the same district. For the last model, we further combined our two registers and trained a combined model. The last three models were tested with 10-fold cross-validation.

### 4.4. Post-Processing

In our problem, we had three different classes: background, individual registers, and separations between regions. Therefore, we evaluated the probabilities of pixels that belonged to one of the classes. For each class, there was a binarized matrix showing the probabilities that a pixel belonged to them. By using these matrices, pixels should be connected, and components should be created. The connected component analysis tool [15] was used for creating objects. After the objects were constructed for all classes, the performance of our system could be measured.

### 4.5. Assigning Individuals to the Populated Places

This toolbox [15] found the objects in all pages by supporting batch processing. However, for our purposes, we needed the number of people in any populated place. To this end, we designed an algorithm for counting people and assigning them to the populated places.

**Figure 6:**
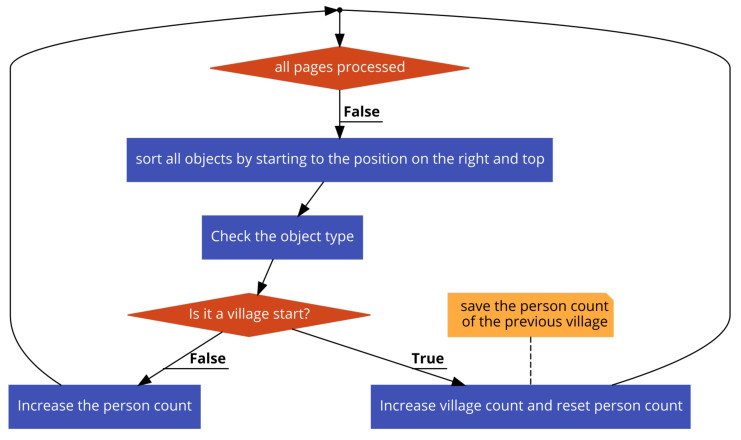
Flowchart of our populated place assigning algorithm.

Firstly, we recorded the x and y coordinates of the rectangles of the found objects. The object could be of a populated place start or individual type. Furthermore, clerks divided each page into two blocks, and we had to consider this structure also. We defined a center of gravity for each object. It was computed by averaging all four coordinates of the rectangle surrounding the object. We used it for comparing the positions of individual objects and populated place start symbols for assigning people (see Algorithm 1 and Figure 6). Due to the structure of the Arabic language, if an object is closer to the top of the page and right of the page compared to any other object, it comes before. However, if the object is in the left block of a page, without looking at the distance from the top, it comes after any object in the right block of the page. We first sorted populated place start objects. For all individual objects, we compared their position on the page and the page number with all populated place start objects. If the individual object was after a populated place start object N and a before populated place start object N + 1, we assigned the individual to populated place N.
**Algorithm 1** The algorithm for assigning individuals into the populated places. Obj. stands for objects, which could be an individual object or a populated place start object. CoG stands for the Center of Gravity of the object.1:**procedure**Compare_Objects(Obj1, Obj2)2:    **if** Obj1.page < Obj2.page **then**3:**return** Obj1, Obj24:    **else if** Obj1.page > Obj2.page **then**5:**return** Obj2, Obj16:    **else if** Obj1.page = Obj2.page **then**7:**return** COMPARE_OBJECT_POSITION (Obj1.position, Obj2.position)8:    **end if**9:**end procedure**10:**procedure**COMPARE_OBJECT_POSITION(Obj1, Obj2)11:    **if** Obj1_CoG.width > Obj2_CoG.width **AND** Obj1_CoG.height < Obj2_CoG.height **then**12:**return** Obj1, Obj213:    **else**14:**return** Obj2, Obj115:    **end if**16:**end procedure**17:**procedure**FIND_POPULATED_PLACE18:    SORT_POPULATED_PLACE_LIST()19:    **for** $individual\_Object_{k}\in \left\{1,\ldots ,K\right\}$ **do**20:        **for** $pop\_place\_start\_Object_{x}\in \left\{1,\ldots ,X\right\}$ **do**21:           COMPARE_OBJECTS ($individual\_Object_{k},pop\_place\_start\_Object_{x}$ )22:           COMPARE_OBJECTS ($individual\_Object_{k},pop\_place\_start\_Object_{x+1}$ )23:           **if** $pop\_place\_start\_Object_{x}$ < $individual\_Object_{k}$ < $pop\_place\_start\_Object_{x+1}$ **then**24:               $individual\_Object_{k}$ BELONG_TO $pop\_place\_start\_Object_{x}$25:           **end if**26:        **end for**27:    **end for**28:**end procedure**

### 4.6. Baseline Heuristic Projection Profile Algorithm for Object Detection

We further implemented the heuristic projection profile algorithm for object detection since the registers had tabular-like layouts. We used the results of this simple algorithm as a baseline and compared it with the CNN-based approach. The heuristic projection profile object detection system is shown in Algorithm 2. As shown in Figure 1, each page had left and right parts, which could have different rows and columns. Therefore, we examined them separately. For each part, horizontal profiles were applied. If there was a minimum of 50 consecutive black pixels between white pixels, a new row was added. The coordinate of rows were recorded with this method. After that, for each row, a vertical profile was applied. Since the objects in a row were closer vertically, we decreased the consecutive black pixel threshold to 20 pixels. The detected object coordinates were recorded. We also needed to distinguish the object types. We classified them by using the area of the object. The area of population start objects was greater than individual count objects. We determined a threshold of 100,000 pixel squares and classified the object as a population start if it had an area greater than the threshold. Otherwise, it was labeled as an individual count object.
**Algorithm 2** Detecting and counting objects belonging to different classes with the heuristic vertical and horizontal projection profiles.1:**procedure**Horizontal_Profile(page_image)2:    **if** 50 Pixels-Long Zero-Pixel Areas FOUND **then**3:        ADD ROW4:    **end if**5:**return** ROWS6:**end procedure**7:**procedure**Vertical_Profile(row)8:    **if** 20 Pixels-Long Zero-Pixel Areas FOUND **then**9:        ADD OBJECT10:        ObjectCount = 20PixelAreaCount +111:    **end if**12:**return** ObjectCount, OBJECTS13:**end procedure**14:**procedure**Calculate_Area(object) **return** AREA15:**end procedure**16:**procedure**Detect_Count_Object_Type(object)17:    AREA = CalculateArea (object)18:    **if** AREA > 100.000 PixelSquares **then**19:        ObjectType = PopulatedPlaceStart20:        PopPlaceStartCount ++21:    **else**22:        ObjectType = Individual23:        IndividualCount ++24:    **end if**25:**return** IndividualCount, PopPlaceStartCount26:**end procedure**27:**for**$page\_image_{k}\in \left\{1,\ldots ,K\right\}$**do**28:    ROWS = HORIZONTAL_PROFILE($page\_image_{k}$)29:    **for** $row_{j}\in \left\{1,\ldots ,J\right\}$ **do**30:        OBJECTS = VERTICAL_PROFILE ($row_{j}$)31:        **for** $object_{i}\in \left\{1,\ldots ,I\right\}$ **do**32:           (IndividualCount, PopPlaceStartCount) = DETECT_COUNT_OBJECT_TYPE ($object_{i}$)33:        **end for**34:    **end for**35:**end for**

## 5. Experimental Results and Discussion

In this section, we first define the metrics used for evaluating our system. We then present our results and discuss them.

### 5.1. Metrics

To evaluate our system performance, we used five different metrics. The pixel-wise classification accuracy, pixel-wise precision, recall, $F_{measure}$, and intersection over union metrics are low-level evaluators, and they are widely used in object detection problems [32]. We also defined high-level counting error metrics to evaluate the accuracy of our system.

#### 5.1.1. Pixel-Wise Classification Accuracy

The first metric is the pixel-wise accuracy. It can be calculated by dividing the accurately classified pixels in each document by the number of all pixels (for all object types). Note that it was calculated for each page and averaged over all pages in the test set.

#### 5.1.2. Pixel-Wise Precision, Recall, and $F_{measure}$

We further calculated pixel-wise True Positive (TP), True Negative (TN), False Positive (FP), and False Negative (FN) metrics for the object detection problem. Note that these metrics were for the two-class classification of objects (individual and populated place start) versus background individual objects versus background and starting symbols versus background. By using these metrics, we calculated pixel-wise precision, recall, and $F_{measure}$ for the object detection problem as:

(1) $$Precision=\frac{TP}{TP+FP}$$

(2) $$Recall=\frac{TP}{TP+FN}$$

(3) $$F_{measure}=\frac{2\times Precision\times Recall}{Precision+Recall}$$

Note that these metrics were calculated for each page and averaged over all pages in the test set.

#### 5.1.3. Intersection over Union

The Intersection over Union (IoU) metric was also calculated. For this metric, there were the ground-truth components and the predicted components from our model. This metric could be calculated by dividing the intersection of regions of these two components by the union of regions of these two components (for all object types).

#### 5.1.4. High-Level Counting Errors

These metrics were specific to our application for counting people in registers. For counting the individuals, the first high-level metric could be defined as the predicted count errors over the ground truth count. We called this metric Individual Counting Error (ICE).

(4) $$ICE=||\frac{PredictedIndividualCount-Ground-TruthIndividualCount}{Ground-TruthIndividualCount}||$$

We further defined a similar high-level metric for populated place start objects, which was named the Populated Place Start Counting Error (PPSCE).

(5) $$PPSCE=||\frac{PredictedPopPlaceStartCount-Ground-TruthPopPlaceStartCount}{Ground-TruthPopPlaceStartCount}||$$

### 5.2. Results and Discussion

#### 5.2.1. Results from the Heuristic Baseline Projection Profile Technique

**Figure 7:**
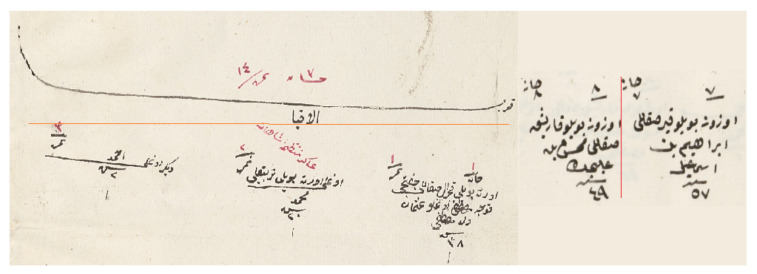
Examples of intertwined rows and columns are shown. They are counted as one since there are not any empty pixels in between.

We first extracted results by using the baseline projection profile technique. After applying horizontal and vertical projection profiles consecutively, we applied an area-based object classification on detected fields. We changed the parameters for Zistovi registers, because they were tightly placed and default parameters caused errors. The error rates are provided in Table 1. The errors were generally caused by intertwined individual objects and closely written individual and populated place symbols (see Figure 7). Since we used an area-based object classifier, when two or more individual objects were counted as one, they were classified as a populated place start symbol since the area of the detected object passed the threshold for object detection. The errors in Nicea registers were higher because individual objects and populated place start symbols intertwined more often.

#### 5.2.2. CNN-Based Page Segmentation and Object Detection

**Figure 8:**
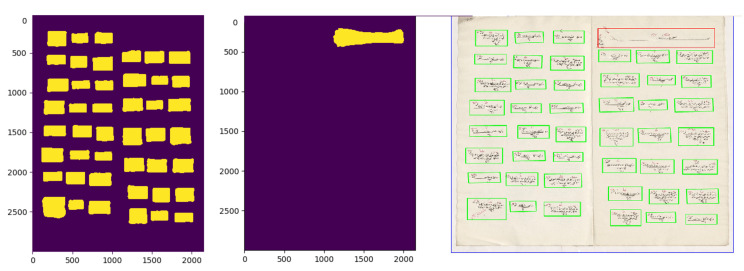
A sample prediction made by our system. In the left, a binarized prediction image for counting individuals, in the middle, a binarized image for counting populated place start, and in the right, the objects, enclosed with rectangular boxes. Green boxes for individual register counting and the red box for counting the populated place start object.

**Figure 9:**
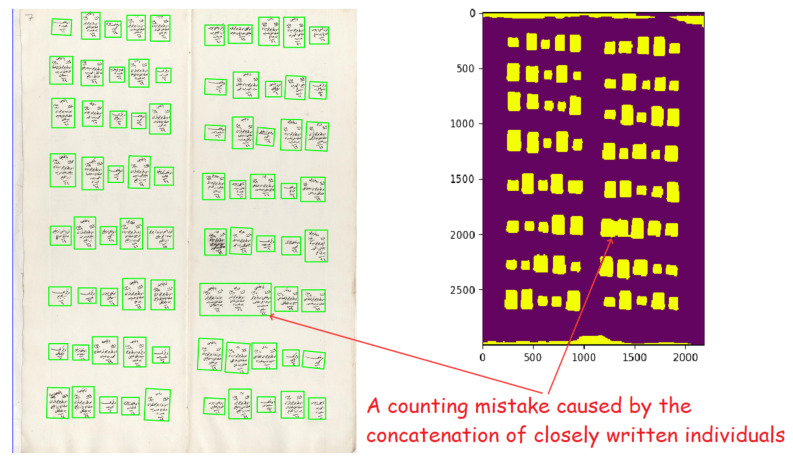
A sample counting mistake. All three individual registers are counted as one. This results in two missing records in our automatic counting system.

We had two registers from the Nicaea district and one register from the Svishtov district. In Model 1, we trained with Nicaea registers and tested with the Svishtov registers. In Model 2, we trained a model with the Svishtov district register and tested with the Nicaea registers. We further tested 10-fold cross-validation on registers in the same district. In Model 3, we trained and tested the model on the Svishtov registers, and in Model 4, we trained and tested on Nicaea registers with 10-fold cross-validation. In Model 5, we combined the whole dataset and evaluated the model with 10-fold cross-validation. The pixel-wise accuracy, IoU, $F_{measure}$, and counting error results are provided in Table 2, Table 3, Table 4 and Table 5. In Table 2, the results for all object types are presented. Note that the combined objects versus background classification results are provided in Table 3, whereas separate objects versus background classification results are provided in Table 4 and Table 5. From these tables, we can see that the individual object detection results were better than the starting symbol detection results. The error of finding the number of individuals and the populated place start objects is provided in Table 2. We further provide correctly predicted and mistakenly predicted raw binarized images in Figure 8 and Figure 9, respectively. The best ICE results were obtained when the Svishtov registers were used for the training. The worst accuracy was obtained when the system was trained with Nicaea registers and tested with the Svishtov register. Furthermore, the populated place start counting error was 0% for all models, which meant that our system could recognize populated place start objects perfectly in the considered experiments.

As mentioned before, the layout of registers depended on the districts and the clerk. For our registers, individuals in Nicaea were widely separated, whereas the distance between registers was less in Svishtov registers. The average number of registered individuals in a Nicaea register page was approximately 40 and 80 in a Svishtov register, which confirmed the above statement. Therefore, when the system was trained with loosely kept Nicaea registers and tested in closely written clusters in Svishtov, the counting error increased, and the number of mistakes for counting multiple registers as one started to occur (see Figure 9). However, if we changed the training and test parts, the system error for counting objects approached 100%, as we expected. If we mixed the dataset and applied 10-fold cross-validation, we achieved counting errors in between. For our purposes, although high-level metrics were more crucial, low-level metrics showed the general performance of our system. They were also beneficial for comparing the performances of different models. Furthermore, even though the IoU metric results were low, our classification errors were close to 0%. It could be inferred that the structure of the registers was suitable for automatic object classification systems. The documents did not have printed tables, but their tabular-like structures made it easier to cluster and classify them.

We further compared our work with the segmentation techniques applied to different historical Arabic script documents. The $F_{measure}$ of our study was slightly higher than the best reported results in the literature (see Table 6). However, because the different techniques were tried in different datasets created for each particular study in the literature, one could not infer the success of a technique over others. They were presented to give the reader a sense about the performance of the object vs. background classification problem in historical Arabic script documents. Having said that, we could state that our results were aligned with the best reported results in the literature for historical Arabic script document layout analysis.

## 6. Conclusions and Future Works

In this study, we developed an automatic individual counting system for the registers recorded in the first censuses of the Ottoman Empire, which were held between 1840 and 1860. The registers were written in Arabic script, and their layouts highly depended on the district and the officer in charge. We created a labeled dataset for three registers and evaluated our system on this dataset. We further developed an algorithm for assigning people to populated places after detecting individual people and populated place start symbols. For counting the populated place start symbols, we achieved 0% error. Furthermore, we achieved the maximum individual counting error of 0.27%. We inferred from these results that the models should be trained with closely placed and noisy registers (Svishtov register in our case study). When these models were tested with a clean and a loosely placed one (Nicaea register in this case study), the system counted individuals accurately. However, if a model was trained with a loosely placed register and tested with a closely placed one, the number of counting errors increased. Our aim was to develop a generic system that could be implemented for efficient counting and distant reading of all registers prepared between the 1840s and the 1860s. Since it is a very costly task to label all registers, we will strategically label the closely placed and noisy ones to develop such a system. As future works, we plan to develop an automatic handwriting recognition system for the segmented individual register objects. We further plan to implement self-organization map and projection profile algorithms to compare with the CNN in our dataset.

## Figures and Tables

**Table 1:** Results with different metrics are presented for five different models. ICE, Individual Counting Error.

Tested with	PPSCE(%)	ICE (%)
Nicaea	10	2.927
Svishtov	3.703	2.412

**Table 2:** Results with different metrics are presented for five different models.

Trained on	Tested with	Pixel-Wise Acc.(%)	IoU (%)	ICE (%)	PPSCE (%)
Nicaea	Nicaea	91.53	80.91	1.65	0
Nicaea	Svishtov	90.35	73.6	11.57	0
Svishtov	Svishtov	93.39	72.29	0.76	0
Svishtov	Nicaea	91.92	47.95	0.27	0
Mixed	Mixed	92.54	48.54	2.26	0

**Table 3:** True positive, true negative, false positive, false negative, precision, recall, and $F_{measure}$ results are presented for the five different models. The results were obtained for combined objects (populated place start and individual objects) versus background.

Trained on	Tested with	TP	TN	FP	FN	Recall	Precision	$F_{\mathrm{measure}}$
Nicaea	Nicaea	0.7463	0.169	0.1367	0.0702	0.913	0.981	0.9459
Nicaea	Svishtov	0.7455	0.158	0.0125	0.0834	0.898	0.983	0.9386
Svishtov	Svishtov	0.7769	0.157	0.0191	0.0469	0.942	0.975	0.9587
Svishtov	Nicaea	0.7822	0.137	0.0596	0.0209	0.974	0.928	0.9499
Mixed	Mixed	0.7824	0.143	0.0379	0.0366	0.955	0.953	0.9539

**Table 4:** True positive, true negative, false positive, false negative, precision, recall, and $F_{measure}$ results are presented for five different models. The results are obtained for populated place objects versus background.

Trained on	Tested with	TP	TN	FP	FN	Recall	Precision	$F_{\mathrm{measure}}$
Nicaea	Nicaea	0.79517	0.18316	0.02138	0.00028	0.88576	0.99862	0.9376
Nicaea	Svishtov	0.80640	0.17583	0.01751	0.00024	0.90168	0.99830	0.9469
Svishtov	Svishtov	0.80089	0.17602	0.02302	0.00005	0.87563	0.99952	0.9319
Svishtov	Nicaea	0.79172	0.19341	0.01381	0.00104	0.91688	0.99518	0.9528
Mixed	Mixed	0.79934	0.18020	0.0204	0.00005	0.88005	0.99969	0.9341

**Table 5:** True positive, true negative, false positive, false negative, precision, recall, and $F_{measure}$ results are presented for five different models. The results are obtained for individual objects versus background.

Trained on	Tested with	TP	TN	FP	FN	Recall	Precision	$F_{\mathrm{measure}}$
Nicaea	Nicaea	0.79458	0.16838	0.01506	0.02199	0.97252	0.98093	0.9766
Nicaea	Svishtov	0.81280	0.17809	0.0902	0.0500	0.94681	0.98859	0.9639
Svishtov	Svishtov	0.80669	0.15349	0.02259	0.17215	0.97867	0.97236	0.9754
Svishtov	Nicaea	0.80168	0.12277	0.07160	0.00385	0.99514	0.91634	0.9537
Mixed	Mixed	0.80000	0.16613	0.01413	0.01970	0.97581	0.98306	0.9794

**Table 6:** Comparison of our results with different studies on Arabic historical document layout analysis. FCN stands for Fully-Convolutional Networks.

Study	Dataset	# of Pages	Classifier	$F_{\mathrm{measure}}$
Bukhari et al. [8] (2012)	Islamic manuscripts in Leipzig	38	MLP	0.9502
Hesham et al. [18] (2017)	DARPA MADCATdataset	10	SVM	0.8514
Barakat et al. [16] (2018)	Islamic manuscripts in Leipzig	38	FCN	0.95
Our study (2020)	Ottoman Population Registers	173	CNN	0.9539 (Mixed)
